# 2340. COVID-19 Vaccine Hesitancy among Faculty Members, College Students and Health Care Workers in Lebanon

**DOI:** 10.1093/ofid/ofad500.1962

**Published:** 2023-11-27

**Authors:** George Doumat, Mohamad Ali Tfaily, Rayyan Wazzi-Mkahal, Zeina Kanafani

**Affiliations:** American University of Beirut, Beirut, Beyrouth, Lebanon; Emory University, Atlanta, Georgia; American University of Beirut, Beirut, Beyrouth, Lebanon; American University of Beirut Medical Center, Beirut, Beyrouth, Lebanon

## Abstract

**Background:**

The COVID-19 pandemic has caused significant morbidity, mortality, and economic losses worldwide. Despite the efficacy of several vaccines in reducing SARS-CoV-2 infections, vaccination hesitancy remains a challenge. This study aimed to examine COVID-19 vaccination perceptions, beliefs, attitudes, and knowledge in Lebanon and identify unique predictors of vaccine hesitancy.

**Methods:**

We conducted a cross-sectional online survey between March and June 2021 at the American University of Beirut and its Medical Center targeting faculty members, students, and healthcare workers. The survey included four sections, covering socio-demographics, COVID-19 perceptions, attitudes, and knowledge, with a primary focus on vaccination intent. We used a multivariate logistic regression model to investigate potential determinants of vaccine hesitancy.

**Results:**

We had 422 respondents with an overall response rate of 94%. A large proportion of the study population were females (69%) and individuals with at least undergraduate college education (93%). Healthcare workers comprised 29.4% of all survey respondents. Of the respondents, 368 (87%) were definitely willing to take the COVID-19 vaccine, and 13% expressed various forms of vaccine hesitancy (1% definitely not willing; 8% undecided likely; 4% undecided unlikely). The most important independent predictors of vaccine hesitancy were the perception that the COVID-19 vaccine is more dangerous that the COVID-19 infection, that the vaccine development was rushed, and being a healthcare worker. Knowledge about COVID-19 infection, level of education, age, and recommendation from the respondent’s healthcare provider were negatively correlated with vaccine hesitancy. The multivariable analysis is shown in the Table.

**Figure:**
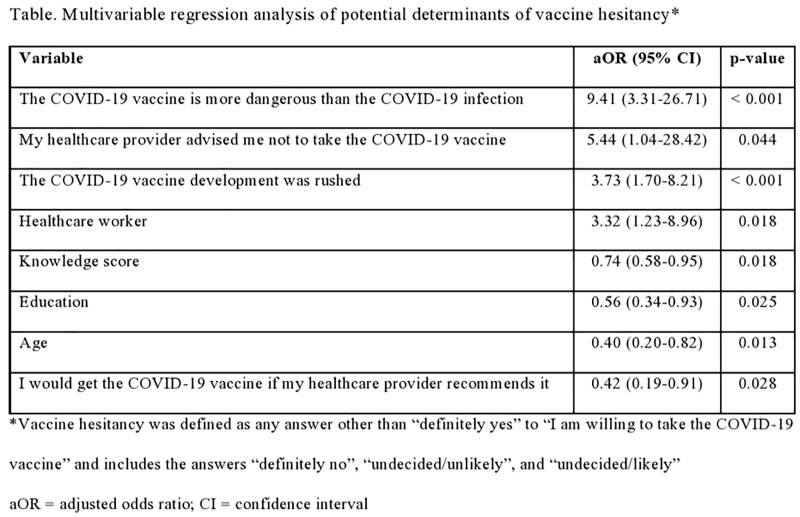

**Conclusion:**

We found a high COVID-19 vaccine acceptance rate and some of the factors associated with hesitancy were modifiable, including knowledge and perceptions about the disease and the vaccine, and healthcare provider recommendation. Addressing these factors is crucial for successful vaccination programs, as combatting vaccine hesitancy may be as crucial as developing a safe and effective vaccine.

**Disclosures:**

**All Authors**: No reported disclosures

